# Effect of E-waste copper alloy additions on the microstructure and organization of Cu90PSn brazing joints

**DOI:** 10.3389/fchem.2024.1342117

**Published:** 2024-03-22

**Authors:** Li Bao, Jiao Yang, Shannan Zhang, Tianran Ding

**Affiliations:** State Key Laboratory of Advanced Brazing Filler Metals and Technology of Zhengzhou Research Institute of Mechanical Engineering Co., Ltd., Zhengzhou, Henan, China

**Keywords:** electronic waste smelting copper alloy, Cu90PSn brazing filler metal, microstructure, joint performance, welding

## Abstract

The effects of different contents of e-waste alloy on the microstructure and joint properties of Cu90PSn brazing filler metal was investigated during copper and copper brazing. Microstructure of base metal and brazing filler metal was studied with scanning electronic microscopy (SEM). The properties of brazing joint obtained by adding different electronic waste filler metal for smelting copper alloy were compared together. The results indicated that the fluidity of Cu90PSn brazing filler metal was weakened and the spreading property of Cu90PSn brazing filler metal was damaged after the addition of e-waste copper alloy. The structure of Cu90PSn brazing filler metal is mainly composed of (Cu), Cu_3_P and (Cu,Sn) compounds. When a small amount of electronic waste copper alloy is added, a trace amount of Fe in the brazing filler metal is distributed in the matrix structure of the filler metal in the form of solid solution. With the increase of copper alloys contents by smelting e-waste, Fe content in Cu90PSn brazing filler metal increases; the granular Fe_3_P phosphide changes into lamellar form. The Cu_3_P compound phase changes from continuous large orderly arrangement to discontinuous small block structure. Therefore, adding a trace amount of electronic waste copper alloy to the solder induction brazing copper/copper can obtain a uniform composition of the brazing structure. And the welding performance is not affected. However, As the content of e-waste smelted copper alloy continues to increase, the tensile strength shows a downward trend, which is attributed to the presence of brittle compound Fe_3_P in the joint.

## 1 Introduction

In the evolving landscape fueled by scientific progression and changing times, there is a surging demand for home appliances and electronic devices. Consequently, these products are being replaced more frequently, invariably leading to a considerable accumulation of electronic waste (e-waste). This e-waste not only instigates severe environmental degradation but also promotes substantial resource wastage. The primary concern in the metal recycling of e-waste currently lies in the retrieval of precious metals, a process which predominantly harvests simple metals and is associated with numerous issues (Jiang et al., 2016; Kang et al., 2020; Rao et al., 2020). E-waste fundamentally consists of plastics, inert oxides, and metals. The latter are significantly found in circuit boards, primarily encompassing common metals such as Cu, Sn, Fe, Ni, Al, and Zn, while also containing smaller quantities of Mo, Sb, and precious metals like Au and Ag (Zhang et al., 2018; Fu et al., 2019; Gu et al., 2019; Park and Kim, 2019). Given the high presence of elements such as Cu, Fe, Sn, Ni, Ag, and Zn in e-waste, theoretically, it is feasible to utilize circuit board alloys as the primary component in the creation of copper-based brazing metals (Kell, 2009; Guo et al., 2015; Hsu et al., 2019; Rene et al., 2021; Murthy and Ramakrishna, 2022; Shahabuddin et al., 2023). Nonetheless, experiments have unveiled that directly melting e-waste to manufacture copper-base alloy for direct usage as brazing filler metal is not viable due to high melting temperatures, unsatisfactory alloying extents, and poor performance of the resulting brazing filler metal (Sun et al., 2016; Yang et al., 2022). Preliminary analyses suggest that this is mainly attributed to the high iron content (4–8 wt %) in the copper alloy derived from e-waste, significantly altering its microstructure and properties (Yang et al., 2022). Therefore, the present study proposes the incorporation of e-waste smelted copper alloy into other copper base brazing alloys as an additive component, aiming for comprehensive resource utilization (Zhang and Shi, 2004). This approach anticipates analyzing the influence of different additive proportions on the microstructure and characteristics of the brazing alloys.

In this paper, the microstructure of Cu90PSn brazing filler metal, the welding microstructure and tensile strength of the butt joint of brazing filler metal, which added electronic waste to smelt copper alloy (hereinafter referred to as copper alloy), were evaluated. We endeavor to scrutinize the impact of melting alloys with varied e-waste contents on the wetting properties, weld forming, and joint features, seeking to identify the optimal addition threshold of the copper alloy in the brazing filler metal. The ultimate goal is to diminish the negative influence of the Fe element on Cu-P brazing metal, facilitating the attainment of superior joint formation principles. This study promises to be a substantial contribution, which provides a significant reference for the utilization and research into e-waste recycled copper alloy in the field of brazing.

## 2 Experiments

### 2.1 Composition design of brazing filler metal

**FIGURE 1:**
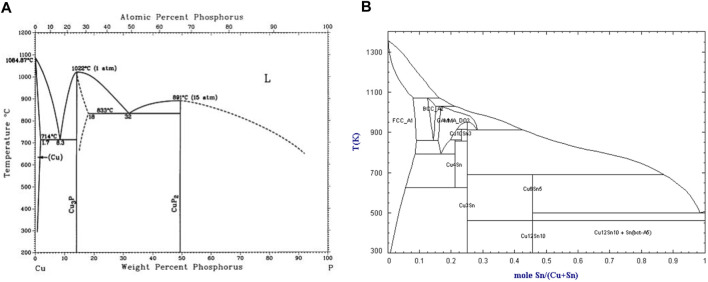
Phase diagram (Tang and Tian, 2009; Zhang and Zhuang, 2011). **(A)** Cu-P binary phase diagram **(B)** Cu-Sn binary phase diagram.

In this study, we opted for the established Cu90PSn brazing filler metal. Drawing upon the Cu-P and Cu-Sn phase diagrams depicted in Figure 1 and corroborated by relevant literature (Zhuang and Sun, 1989; Tang and Tian, 2009; Zhang and Zhuang, 2011; Li et al., 2018), it was discerned that the cast structure of Cu90PSn brazing filler metal predominantly comprises the α-Cu solid solution, the Cu_3_P compound, and a low-melting-point eutectic; noticeably absent was any compound involving tin.

Previous research indicates that the Fe element frequently emerges as a substantial impurity in Cu-based brazing filler metals, particularly in the Cu-P series (Xue et al., 1998; Li, 2018). This intrusion of Fe leads to the creation of a hard and brittle needle-like Fe phase within the microstructure of Cu-P brazing filler metal, thereby compromising its performance and its utility in production applications. Consequently, the study dictates a cap on the e-waste copper alloy addition at 20 wt.% to delve into the repercussions of varying copper alloy amounts on Cu90PSn brazing filler metal, emphasizing the role of impurity Fe content. The chosen copper alloy increments for this investigation are 0, 1 wt.%, 5 wt.%, 10 wt.%, and 20 wt.%, each introduced to the Cu90PSn brazing filler metal through induction melting. Following the melting process, the filler metal undergoes casting and cooling to acquire its form.

Chemical titration techniques facilitated the quantification of the impurity Fe content in Cu90PSn brazing filler metal infused with disparate copper alloy proportions, the results of which are delineated in Table 1. The data vividly illustrates an escalation in Fe integration into the brazing filler metal concurrent with a rise in e-waste copper alloy input.

**TABLE 1:** Fe Content in Cu90PSn filler metal with added e-waste Copper Alloy (wt.%).

Add e-waste melting alloy content	0	1	5	10	20
Fe content	0	0.18	0.57	1.22	2.46

Given the insights derived from the literature, it is plausible that the infiltrating Fe will foster the genesis of a needle-shaped, hard, and brittle Fe3P phase within the Cu-P brazing filler metal (Xue et al., 1998). Nonetheless, it remains a topic of ongoing investigation whether this theoretical conception holds true in practical applications involving Cu90PSn brazing filler metal, necessitating further explorative discourse.

### 2.2 Experimental process

**FIGURE 2:**
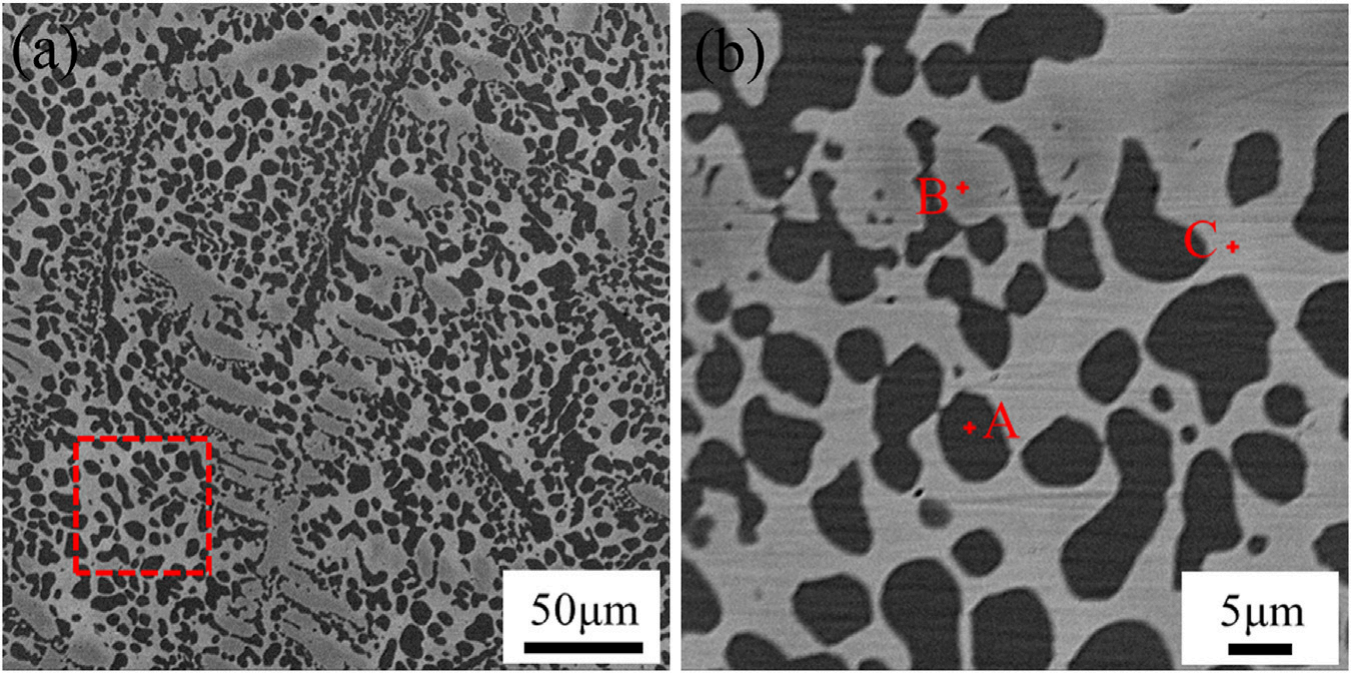
Microstructure of Cu90PSn brazing filler metal without adding e-waste copper alloy **(A)** Cu90PSn brazing filler metal **(B)** Selection area amplification.

Figure 2; Table 2 demonstrated the microstructure and the chemical composition of Cu90PSn brazing filler metal used in the experiments. The addition of e-waste copper alloy to the Cu90PSn brazing filler metal, in proportions of 0, 1%, 5%, 10%, and 20%, preceded the melting and casting of the blend into a block. As inferred from Table 2, the predominant constituents of the resultant Cu90PSn brazing filler metal’s microstructure are Cu-P compounds, the α-Cu phase, and a Sn-rich phase. The microstructure distribution of the brazing filler metal is relatively uniform, which ensures the uniform melting and spreading wetting of the brazing filler metal during brazing.

**TABLE 2:** Results of EDS spectrum analysis at each point in Fig.2(b) (at.%).

Region	Cu	Sn	P	Phase composition
A	68.69	0.08	26.48	Cu-P
B	96.21	1.20	2.59	α-Cu
C	94.79	5.05	0.16	Sn-rich phase

Adhering to the GB/T 11364-2008 standard, we conducted the wetting experiment of brazing filler metal using a pristine copper plate measuring 40 mm × 40 mm × 3 mm. The procedure involved heating the box resistance furnace to the desired temperature before introducing 0.2 g of the brazing filler metal, gauged using a balance, into the furnace for a duration of 1 minute. Subsequently, the material was removed and allowed to cool to room temperature in an aerial setting. The extension of the brazing filler metal was assessed post-cooling. This sequence was replicated thrice, and the mean value of the results documented.

Furthermore, we carried out welding on a purple copper plate using Cu90PSn brazing filler metal infused with varying electronic waste copper alloy contents, extracted from circuit boards. The tensile strength test method of brazing joint delineated in GB/T 11363-2008 guided the tensile tests performed on the welding joints. The tensile strength of joint specimens was tested by universal testing machine. The test was repeated for 5 times, and the average value of the results was taken. To acquire a more detailed understanding, we procured brazing joint samples through wire-cutting, which were then subjected to scrutiny through SEM and Energy Dispersive Spectroscopy (EDS) to analyze the microstructure of the joint sample. This comprehensive approach ensured a robust analysis, providing valuable insights into the microstructural changes and influences induced by varying copper alloy compositions in the brazing filler metal.

## 3 Results and discussion

### 3.1 Wetting properties of brazing filler metal

**FIGURE 3:**
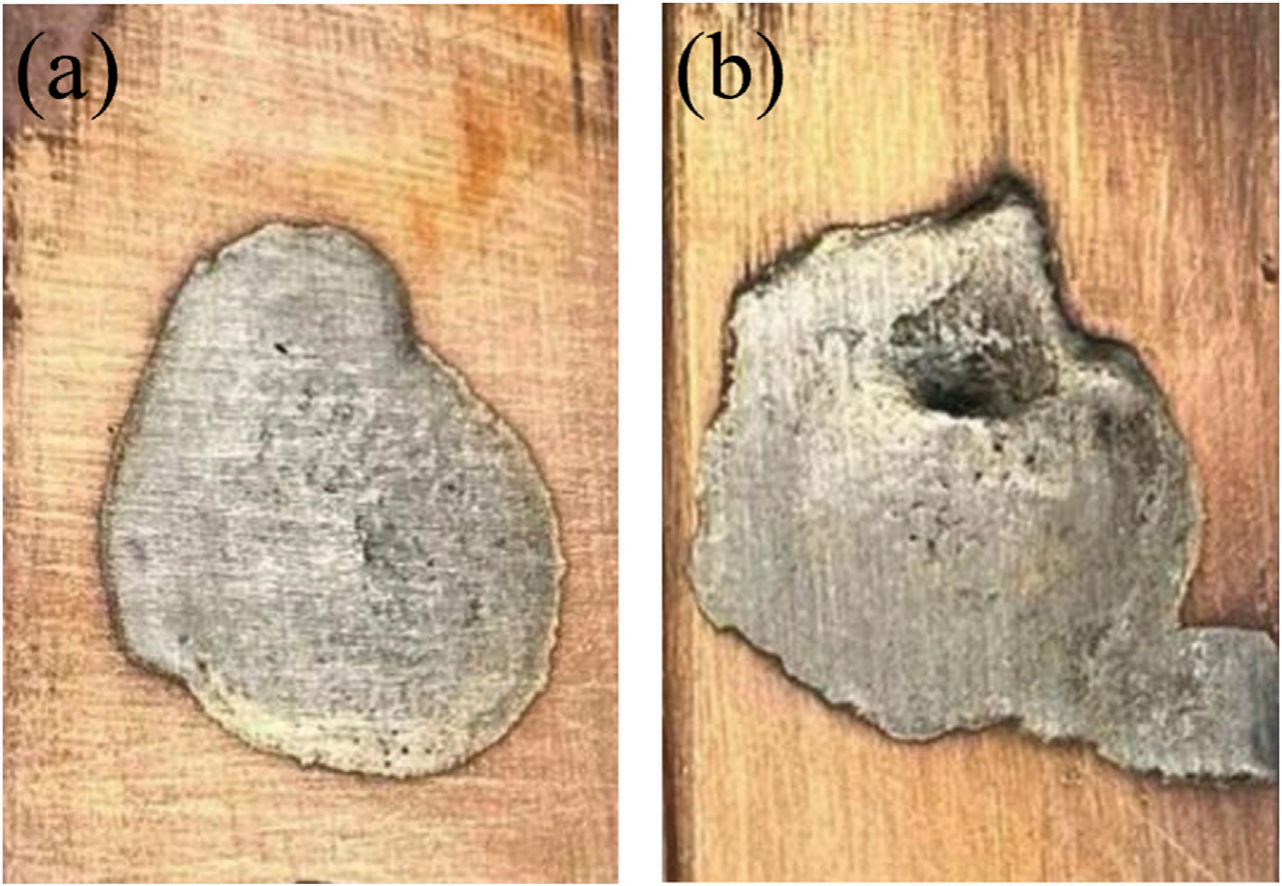
Macrostructure of specimens after wetting experiment **(A)** Cu90PSn brazing filler metal **(B)** Brazing filler metal containing 1 wt.% copper alloy.

**FIGURE 4:**
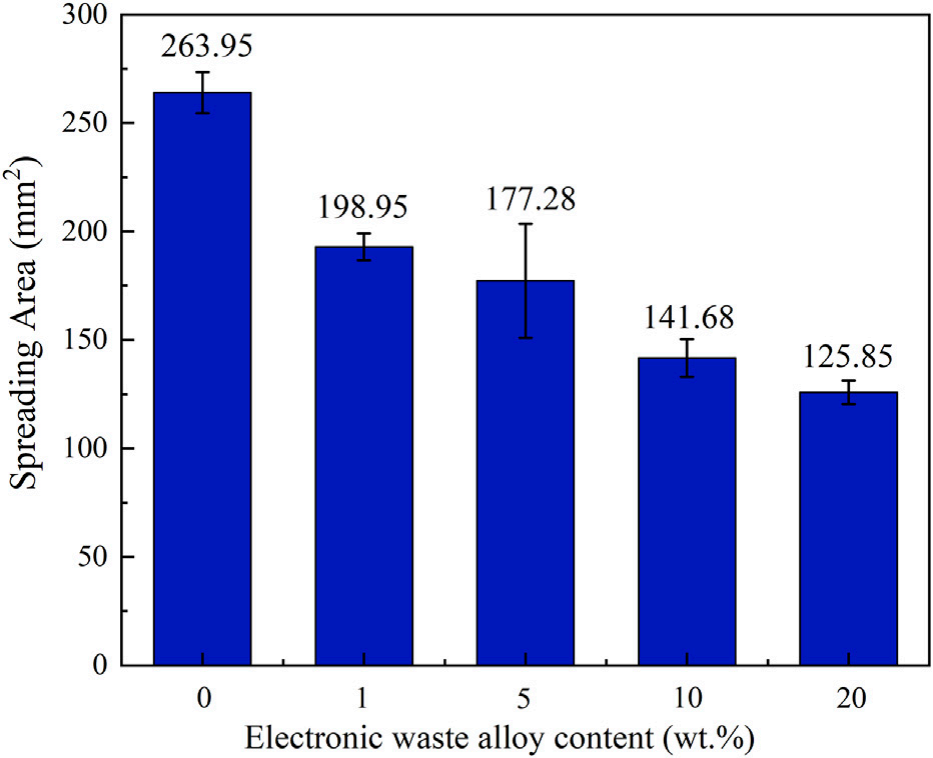
Effect of copper alloy content on the wetting area of Cu90PSn brazing filler metal on copper plate.

Figure 3 delineates the macroscopic morphologies observed in the specimen’s post-incorporation of varying quantities of e-waste copper alloy into the Cu90PSn brazing alloy during the wetting test. The detailed imagery of wetting scenarios across diverse copper alloy concentrations in Cu90PSn brazing alloy was constructed utilizing jigsaw photography facilitated by an in-body microscope; this assisted in measuring the wetting area, the outcomes of which are graphically represented in Figure 4.

As Figure 4 elucidates, the wetting area of Cu90PSn brazing filler metal on the copper-zinc plate exhibits a diminishing trajectory following the incorporation of disparate copper alloy quantities. The zenith of wetting area is attained at 263.95 mm^2^ when devoid of any e-waste copper alloy additives on the copper-violet plate. The scenario alters with 1 wt.% copper alloy supplementation, escalating the iron concentration in the Cu90PSn brazing filler metal, which in turn heightens the melting temperature of the material, thereby reducing the wetting and spreading dimensions to 198.95 mm^2^. A progressive augmentation of e-waste copper alloy to 10 wt.% perpetuates this decline in wetting area, with Fe content reaching 1.22 wt.% and restricting the spread to sub-150 mm^2^ dimensions. This trend substantiates the notion that escalating e-waste copper alloy proportions induce a rise in the Fe content and melting temperature of the brazing filler metal, consequently impeding its wetting efficacy and continually narrowing the wetting area.

The predominant determinants influencing the wetting efficiency of brazing filler metal encompass the compositions of both the brazing alloy and base materials, temperature parameters, the presence of metal oxides, and the surface condition of the base material. Given a constant setting for other variables, the composition of the brazing filler metal prominently impacts the wettability. The integration of electronic waste copper alloy facilitates the ingress of a substantial amount of Fe elements into the Cu90PSn brazing filler metal. In such a scenario, Fe maintains a chemically active free state, predisposing it to facile reactions with oxygen to birth oxides with heightened melting points. Coupled with the presence of P elements in Cu90PSn brazing filler metal, the air-cooling process fosters the development of phosphides, characterized by P atoms occupying the atomic voids in Fe, preserving the structure delineated through metallic bonds. This formulation remarkably augments the viscosity of the brazing alloy, thus escalating the surface tension observed between the brazing and base materials. Such alterations hinder the fluid dynamics of the brazing filler metal during the process, obstructing optimal wetting outcomes.

The gradual elevation in copper alloy concentrations entails not only the central Cu elements but also beneficial constituents such as Sn, Ag, and Ni. However, it also introduces Fe elements to the Cu90PSn brazing filler metal, predisposing it to facile oxidation during preparation and handling. Once the copper alloy addition breaches the 5 wt.% threshold (concomitant with Fe content exceeding 0.2 wt.%), the genesis of brittle phosphides including Fe_2_P and Fe_3_P is observed. This incites the manifestation of along-crystal fractures, debilitating the wetting capabilities of the brazing filler metal. Therefore, while minor inclusions of copper alloy in Cu90PSn brazing filler metal minimally impair the wetting efficacy, surges beyond a 10 wt.% copper alloy content (with Fe content exceeding 1 wt.%) severely undermine the wetting attributes. This, by extension, compromises the brazing performance of the material, introducing adverse implications for its function.

### 3.2 Microstructure and strength of brazing joints

#### 3.2.1 Macro morphology of brazing joints

**FIGURE 5:**
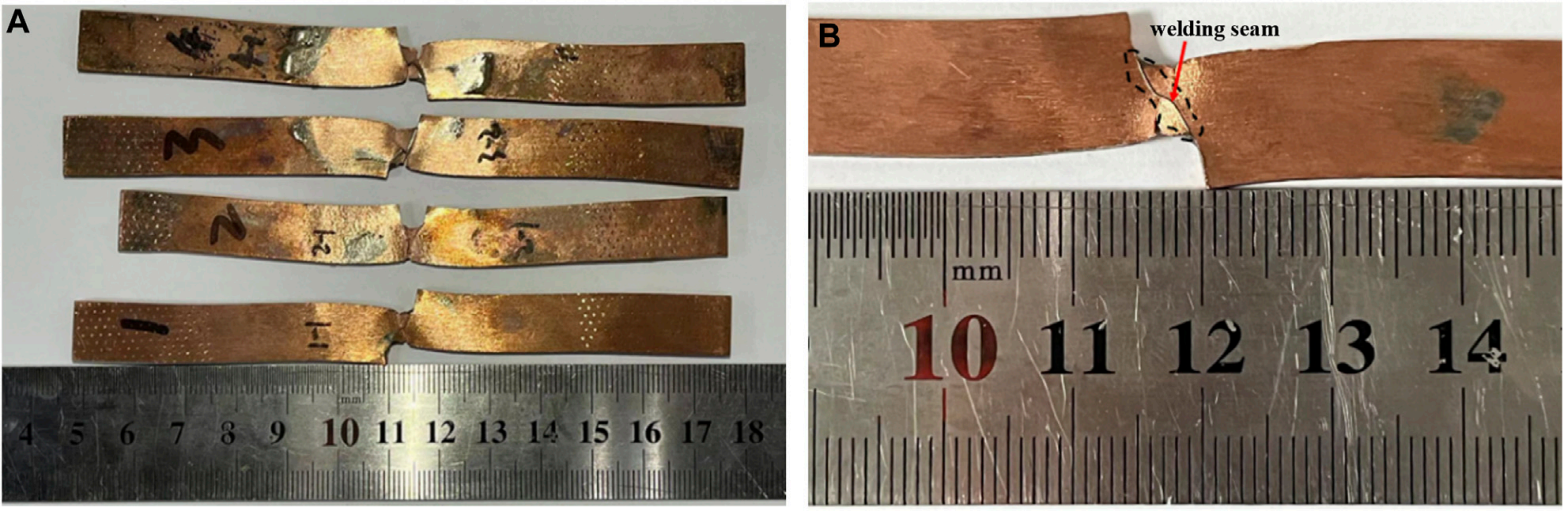
Macroscopic morphology of the joint specimen after tensile test **(A)** Cu90PSn brazing joint with different content of copper alloy **(B)** Local magnification of a single brazing joint.

Figure 5 illustrates the macroscopic morphology of brazing joint specimens following tensile testing, showcasing the fracture patterns. Specimens 1 to 4 utilized Cu90PSn brazing alloys with e-waste copper alloy increments of 0, 1 wt.%, 5 wt.%, and 10 wt.%, respectively, maintaining a joint thickness of 0.3 mm. From Figure 5A, it is discernible that the joints did not fracture entirely post the tensile test, undergoing noticeable torsional deformation in the brazing region. This effect also translates to a distinct elongation of the macroscopic dimensions, extending from an initial 120.15 mm to approximately 130 mm, a phenomenon supported by literary references (Zhuang and Sun, 1989; Zhang et al., 2017). This considerable elongation, reaching over 6.5 mm, indicates a preliminary yield preceding the ultimate fracture. Turning attention to Figure 5B, despite the minimal thickness of brazing layer, residing below 0.15 mm, the primary locus of deformation is the base material substrate, not showcasing any brazing filler metal at the fracture point. This observation signals that the substrate experiences yielding at or below 290.0 MPa, highlighting its inferior yield strength relative to tensile strength of the brazing joint. Consequently, this underscores the superior strength of the brazing filler metal in comparison to the base material, certifying the potential of achieving high-strength brazing joints through the application of this brazing filler metal in welding copper processes.

#### 3.2.2 Microstructure of the brazing joints

**FIGURE 6:**
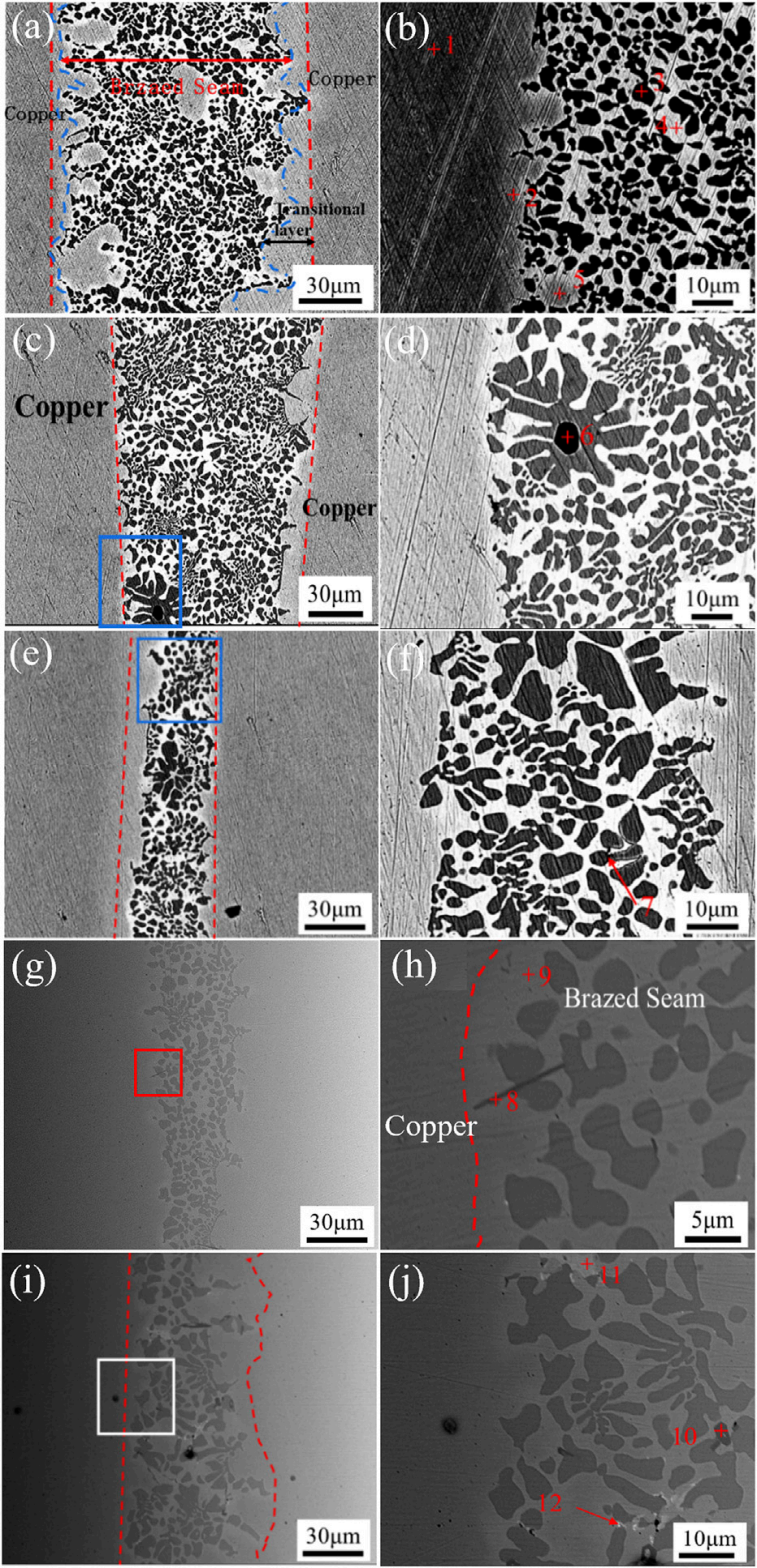
Microstructure of Cu90PSn filler metal brazing joint added different contents of circuit board melting alloy content as shown in **(A)** 0 wt.%, **(C)** 1 wt.%, **(E)** 5 wt.%, **(G)** 5 wt.% and **(I)** 5 wt.%; **(B)**, **(D)**, **(F)**, **(H)** and **(J)** are zoomed in on selected areas of the tissue sites of **(A)**, **(C)**, **(E)**, **(G)** and **(I)**, respectively.

Induction brazing of copper/copper was undertaken using Cu90PSn brazing filler metal with varying concentrations of added copper alloy. The joint microstructures were scrutinized through line scanning, illustrating the tissue morphology of the brazing joints alongside the detailed distribution of the elements, which are elucidated in Figure 6; Table 3.

**TABLE 3:** Results of EDS energy spectral analysis of points in Figure 6 (at.%).

Region	Cu	P	Sn	Fe	Ni	Pb	Ag	C
1	96.45	—	—	—	—	—	—	3.55
2	96.41	2.19	1.39	—	—	—	—	—
3	68.35	26.78	0.64	—	—	—	—	4.23
4	94.02	0.38	5.6	—	—	—	—	—
5	96.13	2.01	1.86	—	—	—	—	—
6	91.64	—	8.36	—	—	—	—	—
7	24.09	33.27	2.87	39.77	—	—	—	—
8	33.9	25.16	1.19	38.27	1.37	—	0.11	—
9	90.86	2.1	5.54	0.48	—	0.44	0.58	—
10	7.97	32.53	0.47	55.85	2.99	—	0.19	—
11	78.1	0.72	18.65	0.23	0.8	—	1.5	—
12	55.02	9.96	10.31	—	0.2	—	24.51	—

Figure 6A delineates the microstructural morphology of the Cu90PSn brazing filler metal when forging copper-to-copper butt joints without the introduction of e-waste copper alloy. This figure elucidates a dense and harmoniously unified joint structure, characterized by smooth brazing seam and evenly distributed welding layer width hovering around 100 μm. Importantly, the interface area is devoid of prominent defects such as inclusions, pores, or cracks, signaling good joint performance and a favorable interaction between the parent copper alloy and the Cu90PSn brazing filler metal during the test. An enlarged view provided in Figure 6B further dissects the joint structure within the welding zone, highlighting the primary constituents: bright white, light gray, and gray-black phases. Synthesizing the insights drawn from the energy spectrum analysis of pertinent zones within the joint structure, displayed in Table 3, deeper understanding emerges. The analysis recognizes region 1 as housing the zinc-copper parent material organization, with the bright white area in the brazing layer being a Sn-rich phase, and the gray-black portion being Cu-rich phase. This interface reveals a narrowly confined transition region embodying dark black small tissue lumps, bright white, and light gray tissues. Furthermore, the table underscores the compositions of regions 2 to 4, detailing a range from the α(Cu) solid solution found in region 2, to distinctive elemental phases spotted in the other regions, establishing the individual roles and composite actions in the resulting structure. Region 3 for the first precipitation of Cu_3_P phase, in the brazing for the black “bean spot” organization; region 4 of the bright white phase contains 10 wt.% of the Sn element is the (Cu)-rich phase.

Venturing into 1 wt.% addition of e-waste copper alloy in the Cu90PSn brazing seam, Figures 6C, D offer a view of the joint structure, which maintains dense and generally even brazing. Noteworthy is the preserved integrity without the presence of slag or porosity and other macroscopic flaws, albeit with a slight reduction in the thickness of welding layer to a range between 70 and 80 μm. Yet, the performance remains satisfactory. A closer examination as presented in Figure 6D reveals the primary constituents in the brazing seam area to be white and gray-black phases. Integrating the data from Table 3 and aligning it with the related literature (Li, 2018), the black lumpy areas are discerned as inclusion phases. Concurrently, inspection of the brazing seam region identifies the dendritic distribution of α-phase primary crystals, paired with the formation of (α + δ) co-precipitates and minor presence of (α + δ + Cu_3_P) eutectic crystals, lending depth to our understanding of the composition and potential performance of these added materials.

Figures 6E–I illustrate the micro-morphology of the brazing joint with the incorporation of 5 wt.%, 10 wt.%, and 20 wt.% copper alloy, respectively. Observing Figure 6F, it becomes apparent that, compared to the Cu90PSn braze microstructure with no added copper alloy, the uniformity of the braze microstructure deteriorates and the brazing layer narrows to span of 20-30 μm upon the addition of 5 wt.% copper alloy. Apart from the primary distribution of white and gray-black phases, small clusters of black phase tissue, interspersed in the gray-black tissue gaps, emerge, constituted of Cu24.09P33.27Sn2.87Fe39.77 (at.%). This is inferred to be composite microstructure of Fe_3_P, Cu_3_P, and α-Cu solid solutions. The arising Fe_3_P phase is predominantly localized near the brazing-base metal interface, exhibiting irregular distribution. A further introduction of 10 wt.% copper alloy exacerbates the loss of braze microstructure uniformity, considerably diminishing the thickness of the transitional layer. The delineation between the brazing seam and the base material becomes less distinct, giving rise to the appearance of needle-like black tissues characterized as the Fe_3_P compound phase. With an escalation in e-waste copper alloy content to 20 wt.%, depicted in Figure 6H, I, the fine granular Fe_3_P compounds undergo transition to lamellar microstructure, persistently occupying the gaps within the gray-black Cu_3_P phase. This evolution fosters a more refined weld tissue with brazing layer thickness fluctuating between 10 and 40 μm, marked by highly irregular tissue distribution. In this context, the Fe and P elements from the e-waste copper alloy amalgamate, curtailing the presence of bean-like Cu_3_P phase. Concurrently, a minor appearance of inclusions within the weld is noted, albeit without the manifestation of cracks.

**FIGURE 7:**
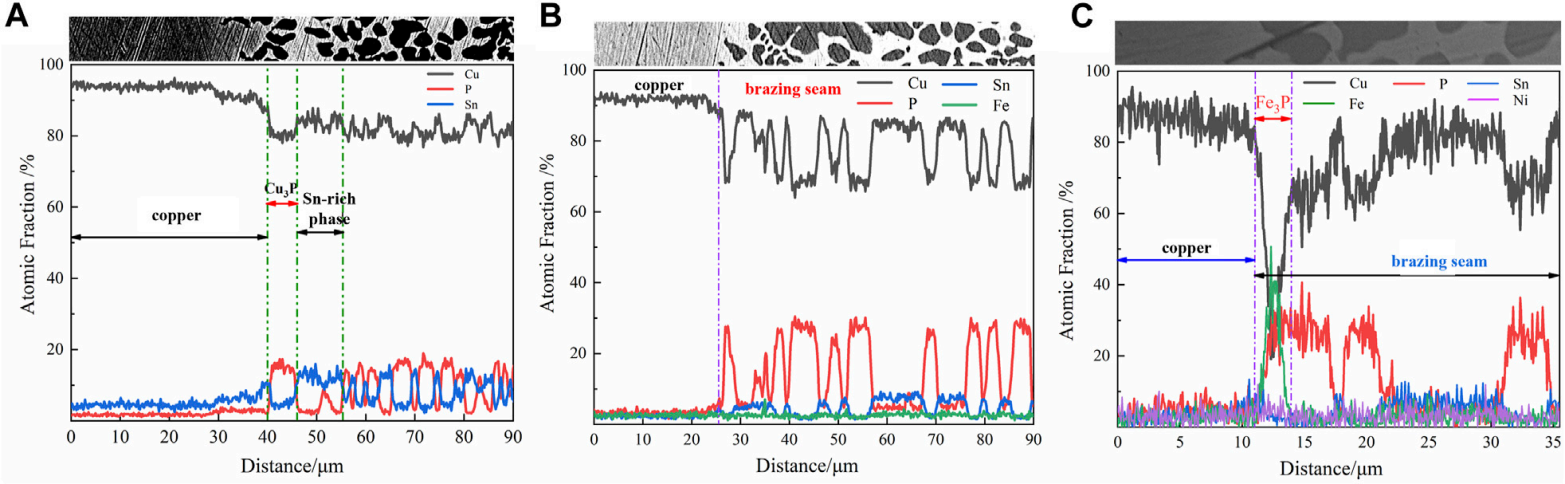
The line scan pattern of Cu90PSn brazing joints corresponding to added different copper alloy content are shown as **(A)** 0 wt.% **(B)** 1 wt.% **(C)** 10 wt.%, respectively.

Figure 7 illustrates the line scan analysis of the elemental distribution across the brazing joints created using Cu90PSn brazing filler metal, encompassing samples with 0, 1 wt.%, and 10 wt.% e-waste copper alloy; spanning from the base material and transition layer to the brazing zone, highlighting the elemental diffusion taking place between the brazing seam region and the adjacent base material. In Figure 7A, pertaining to the joint without added e-waste copper alloy, prominent transitional phase materializes at the interfacial region between the weld and the base material. This illustrates substantial Cu element diffusion from the base material side towards the brazing filler metal side, contrasted with a short-range diffusion of Sn elements migrating from the brazing seam layer to the base material. Noticeably, no diffusion of P elements is observed. Following the introduction of 1 wt.% e-waste copper alloy, as delineated in Figure 7B, Fe elements emerge within the joint microstructure without initiating the formation of brittle iron-phosphorus compounds. Within this construct, Fe elements diffuse towards the base material while Sn and P elements exhibit short-range diffusion from the weld towards the base material, facilitating more even distribution of P elements across the entire joint area. With the addition of 10 wt.% e-waste copper alloy, depicted in Figure 7C, substantial alterations occur in both the joint microstructure morphology and its corresponding line scan. Here, Ni elements from the brazing seam extend their reach into the base material. Meanwhile, surge in the Fe element concentration is noted at the transition layer, countered by dip in Cu content. This region harbors P elements at levels comparable to those forming Cu_3_P, leading to the genesis of Fe_3_P compound with restrained diffusion into the base material. Consequently, the vicinity near the base material is nearly devoid of P elements, and maintains minute Fe content. The transition layer reveals subdued delineation and significant thinning down to non-uniform thickness of merely 25 μm.

#### 3.2.3 Tensile strength of the brazing joints

**FIGURE 8:**
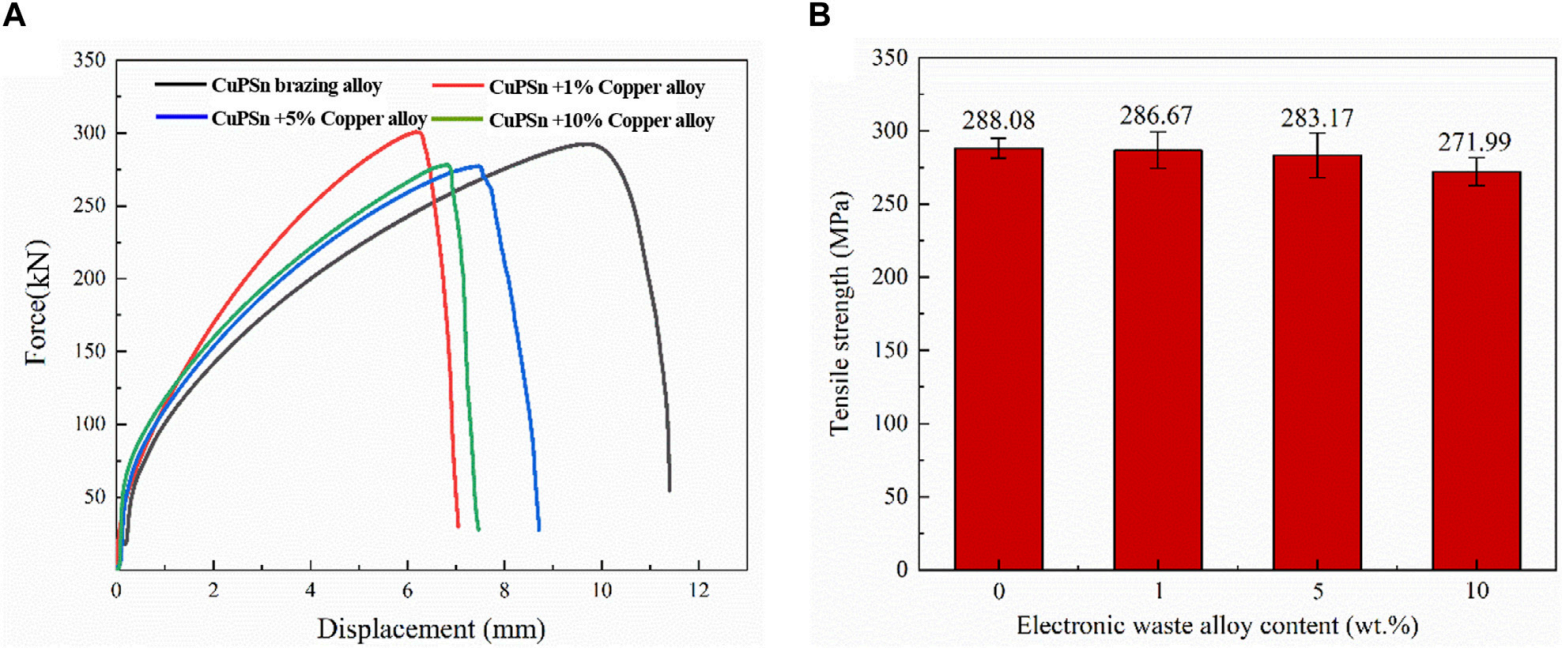
Tensile strength of Cu90PSn brazing joints with different contents of copper alloys **(A)** Forced-displacement curve **(B)** Average tensile strength.

Figure 8 presents the impact of varying e-waste copper alloy concentrations in Cu90PSn brazing alloy on the tensile strength of copper/copper brazing joints, alongside the outcomes depicted through load-displacement curves. According to the load-displacement curves in Figure 8A and corroborated by Figure 5, it is discernible that fractures predominantly manifest on the side of the base material upon the conclusion of the test, phenomenon attributable to the superior strength of Cu90PSn brazing alloy and the annealing and softening of copper during the soldering phase. During the tensile testing of the butt joint, the joint undergoes not only elastic deformation but also a noteworthy extent of plastic deformation, progression validated by the load-displacement curve exhibited in Section 3.2.1. This curve unequivocally demonstrates the onset of substantial deformation prior to fracture in the brazing joints. Given that the base material possesses lesser strength compared to the brazing filler metal, it can be inferred that the main source of this plastic deformation is the base material. This underscores the enhanced mechanical properties endowed to the brazing copper joints by employing Cu90PSn brazing filler metal.

Figure 8B reveals that the incorporation of 1 wt.% e-waste copper alloy into the Cu90PSn brazing filler metal culminated in minor decrement in the average tensile strength of the welding joints to 286.7 MPa, a reduction that, while discernible, does not significantly influence the overall strength. This may be the addition of a small amount of electronic waste copper alloy, the content of less Fe is evenly distributed in the copper solid solution matrix, and no new Fe-containing compounds are formed. At the same time, Fe element has certain diffusion ability and can play a napping role in copper solid solution. As a result, there is no reduction in the tensile strength of the joint. As the concentration of e-waste copper alloy escalates, downward trend in tensile strength is witnessed, accompanied by more pronounced fluctuations in the strength values. When the alloy content ascends to 5 wt.%, a dip in tensile strength to 283.2 MPa is recorded, falling short of the 288.1 MPa achieved without the e-waste copper alloy supplementation. A scrutiny of the brazing interface morphology depicted in Figure 6 elucidates the emergence of diminutive Fe_3_P phases in the interstices of the densely patterned mesh formed of α-Cu and Cu_3_P, evincing gray-black hue. Despite the inception of fragile phosphides in minimal amounts and their considerable separation distances, they theoretically furnish fracture initiation points amidst amplified external forces. Nevertheless, the sizable gaps surrounding the Fe_3_P phases deter swift crack propagation, resulting in only modest decline in strength. Conversely, upon elevating the e-waste copper alloy percentage to 10 wt.%, drastic elevation in brazing temperature transpires alongside marked decline in the tensile strength of the brazing joints. This occurrence can be traced back to the augmented Fe element content deriving from higher ratio of e-waste copper alloy in the Cu90PSn brazing filler metal. During the brazing process characterized by a diminishing temperature regime, Fe collaborates with P to spawn extensive, lamellar, brittle phosphate phases predominantly concentrated near the juncture of the brazing seam and the base material. This phenomenon elevates the brittleness of the brazing metal, compromising the mechanical integrity of the brazing joint by fostering a degradation in its mechanical attributes.

## 4 Conclusion

The following conclusions were obtained by studying the effect of e-waste copper alloy on the microstructure and mechanical properties of Cu90PSn brazing joints:

(1) With the increase of copper alloy content, Cu90PSn brazing filler metal will generate Fe_3_P and other brittle phosphide, significantly reducing the wetting properties of the brazing material. It will also lead to decrease in the tensile strength of the brazing joints. As the e-waste copper alloy content increases to 5 wt.%, the thickness of the brazing seam layer becomes narrower, and a mixed organization of Fe_3_P, Cu_3_P and Cu solid solution appears. When the content of copper alloy in e-waste increased to 5 wt. %, great changes have taken place in the microstructure. These changes refer to the narrowing of the thickness of the brazing seam on the one hand, and the mixed structure of Fe_3_P, Cu_3_P and Cu solid solution on the other hand. With the further increase of copper alloy content in Cu90PSn brazing filler metal, the Fe content in brazing alloy increases. Therefore, the content of Fe_3_P compounds in the microstructure of the solder increases, and the distribution of the weld structure is extremely uneven, which leads to the obvious oxidation phenomenon. Continuing to increase the content of copper alloy. With the Fe content in the solder exceeds 2 wt.%, the filling ability of the brazing alloy is greatly reduced, which causes a sharp deterioration of the welding performance.
(2) In summary, the addition of trace amounts of electronic waste copper alloy (≤1 wt.%) in Cu90PSn brazing filler metal basically does not affect the brazing performance of the brazing alloy by comparing the various aspects of performance analysis of Cu90PSn brazing filler metal with different contents of electronic waste copper alloy. Consequently, the addition of electronic waste copper alloy content of Cu90PSn brazing filler metal should not exceed a maximum of 5 wt.%.

## Data Availability

The original contributions presented in the study are included in the article/Supplementary material, further inquiries can be directed to the corresponding author.
